# Parents’ and Health Care Professionals’ Perspectives on Prevention and Prediction of Food Allergies in Children: Protocol for a Qualitative Study

**DOI:** 10.2196/41436

**Published:** 2023-03-22

**Authors:** Madlen Hörold, Christian Apfelbacher, Katharina Gerhardinger, Magdalena Rohr, Maria Schimmelpfennig, Julia Weigt, Susanne Brandstetter

**Affiliations:** 1 Institute of Social Medicine and Health Systems Research Medical Faculty Otto-von-Guericke University Magdeburg Magdeburg Germany; 2 University Children's Hospital Regensburg (KUNO) Hospital St Hedwig of the Order of St John University of Regensburg Regensburg Germany

**Keywords:** parents, health care professionals, content analysis, grounded theory, food allergy, children, allergic reaction, information needs, information seeking, prevention, prediction, risk factors

## Abstract

**Background:**

Food allergy in children is increasing in prevalence in the western world and appears to become an important health problem. Parents of children at risk of food allergy live with the fear of allergic reaction, especially when the children are very young. The paradigm shift in allergy prevention in the last decade—away from allergen avoidance toward a tolerance induction approach—challenges both parents and health care professionals, as they have to deal with changing information and new evidence that often contradicts previous assumptions. Yet, research on health information–seeking behavior and needs of parents on primary prevention of food allergy in children as well as on prediction and prevention strategies of German health care professionals is lacking.

**Objective:**

The aim of the study is to explore and understand parents’ and health care professionals’ perspectives on the prediction and prevention of food allergies. We are particularly interested in information needs, information seeking, and health care usage and place a special focus on families’ experiences when their child is at risk or diagnosed with food allergies. Furthermore, food allergy prediction and prevention strategies of health care professionals will be explored.

**Methods:**

This study is part of the NAMIBIO (food allergy biomarker) app consortium, which aims to identify early predictors for the development of food allergy in children and develop apps to guide health care professionals and parents of children with a high risk of food allergy toward prevention and timely tolerance induction. The study uses a qualitative approach with topic-guided interviews and focus groups with parents of children (0-3 years) and health care professionals. Data collection will continue until theoretical saturation is reached. The qualitative content analysis will be used according to Kuckartz to identify overarching themes toward information needs and seeking behavior as well as usage of health care and health care professionals’ predictive and preventive strategies. In addition, a constructivist grounded theory approach will be used to explore and understand parents’ experiences, interactions, and social processes in families in daily life.

**Results:**

Recruitment and data collection started in February 2022 and is still ongoing.

**Conclusions:**

The qualitative study will provide insight into parents’ information-seeking behavior and needs regarding the prevention of food allergy in children, parents’ use of pediatric primary care, and health care professionals strategies for the prediction and prevention of food allergies in children. We assume that our results will highlight the challenges associated with the paradigm shift in allergy prevention for both parents and health care professionals. The results will be used to make practical recommendations from the user’s perspective and inform the development of the NAMIBIO apps.

**International Registered Report Identifier (IRRID):**

DERR1-10.2196/41436

## Introduction

### Background

Food allergies (FAs) are an important public health issue affecting children and adults and have been increasing in prevalence in the last 2 to 3 decades [1-3]. The estimated prevalence of food allergy in children (FAIC) in the western world is around 6%-8% [4]. The symptoms can vary from mild to severe, and in extreme cases, FA can lead to a life-threatening allergic reaction (anaphylaxis) [1]. Therefore, it is important to identify children at increased risk before the onset of clinical symptoms and prevent the development of FA. Genetic, epigenetic, and environmental risk factors are increasingly being clarified. This offers a potential for improved prevention and treatment strategies for at-risk groups and possibly all children [2,5]. Insights on pathophysiology reveal a complex interplay of the epithelial barrier, mucosal and systemic immune response, route of exposure, and microbiome among other influences resulting in allergy or in tolerance [6].

Currently, there is no cure for FAs [2]. Avoidance of allergens is the mainstay of management, along with patient education and provision of emergency medication (adrenaline auto-injectors) [2,7]. Prevention approaches based on new research highlight that a strict diet avoiding allergenic foods such as milk, egg, fish, or nuts is not a sensible allergy prevention intervention during pregnancy, breastfeeding, and complementary feeding. The aim is rather to introduce potentially allergenic foods at an early stage in order to promote tolerance induction [4,8].

This paradigm shift in allergy prevention—away from allergen avoidance toward a tolerance-induction approach [8-10]—challenges both parents and health care professionals (HCPs) as they have to deal with changing information and new evidence that often contradicts previous assumptions. Thus, different knowledge of (changing) guidelines, attitudes, and beliefs about FA may result in heterogeneous counseling practices [11] and prevention strategies [12] in pediatricians.

In Germany, pediatricians and midwives are an important source of information on newborn care and allergy prevention for parents [13]. However, parents also seek information from other sources, for example, websites and social media, which can lead to confusion when deciding which recommendation to follow [14,15]. In particular, those with limited access and information-seeking skills, as well as a lack of critical evaluation skills, have been identified as being disadvantaged when searching for health information on the internet [16,17].

Previous studies from other countries (the United Kingdom, Canada, and Australia) showed that parents’ information needs regarding FA are often not met [18-20]. Hu et al [20] found that information needs and information-seeking behavior of parents with a child diagnosed with FA changed over the course of the disease. In particular, in the period after the diagnosis, parents wanted extensive information [20]. Other studies identified a strong parental need for recommendations on how to manage FA and on how to cope with fear and anxiety associated with the disease [18,19].

Chang et al [12] studied the beliefs and practices of general pediatricians from the United States regarding early peanut introduction. Physicians perceived important barriers to the implementation of the recommendations of the guideline. Among other things, they did not fully agree with the content of the guideline and also anticipated that parents would be skeptical. This corresponds to surveys from the United States, Brazil, and Canada, which revealed that awareness and implementation of guidelines on FA prevention varied widely among physicians [21-23].

Until now, there is no research on information-seeking behavior and needs of parents regarding the primary, early childhood prevention of FA as well as on FA prediction and prevention strategies of German HCPs.

### Objectives

The objectives of the study are to (1) identify, conceptualize, and systematically describe (a) parental information needs, information seeking, and health care usage regarding prediction and prevention of FAIC and (b) HCPs’ strategies on prediction and prevention of FAIC and (2) explore and understand how parents experience family life with children at risk of FA or with an existing FA.

## Methods

### Research Context

The study is a subproject of the Nahrungsmittelallergie biomarker (NAMIBIO) app consortium, funded by the German Federal Ministry of Education and Research (01EA2108A-E). The consortium aims to identify early predictors for the development of FAIC and to develop 2 applications to guide HCPs and parents of children at high risk of FA toward prevention and timely tolerance induction. NAMIBIO is a German acronym for Nahrungsmittelallergie biomarker (“food allergy biomarker”). The planned NAMIBIO digital health apps, “Parent app” and “Professional app,” will provide an algorithm for individual prediction of children’s FA risk and help parents of children at high risk of FA and their HCPs to manage their information needs and prevention efforts [24]. While there is an increasing availability of mobile health technology that focuses on FA management [25], to the best of our knowledge, until now, there is no app that focuses on the prediction and prevention of FAIC.

The results of this study will inform the development of the NAMIBIO apps.

### Research Team and Reflexivity

Our subproject is jointly led and conducted by members of the Otto von Guericke University Magdeburg, Faculty of Medicine, Institute of Social Medicine and Health Systems Research and the University Children’s Hospital Regensburg (KUNO) at the St. Hedwig Clinic, Hospital of the Order of St. John, University of Regensburg.

The project team consists of 2 leads (CA and SB), 4 research assistants with master’s degrees (Public Health, Psychology, Sociology, and Social Work in the Aging Society, female), 1 postdoctoral researcher (Nursing Science, female), and 2 student research assistants (female). All team members have previous research experience and training in conducting qualitative research. In order to develop a common understanding of qualitative methods, we shared our prior qualitative research experience in a joint workshop during the elaboration of the study protocol. In this process, interviewing skills were further discussed and practiced as well as prejudices and preassumptions with regard to recruitment, data collection, and results were exchanged and recorded.

We are aware that qualitative research interviewers influence the interaction of the interviews. As interviewers, we want to display self-confidence in what we do, trust in the interview situation and what the participants will share with us, as well as inner tranquility that communicates interest and attention [26]. The participants will be informed about the reasons and aims of this study. We will also inform them that we (the interviewers) are part of the research team and where we are located (institute or university). We will not actively disclose personal information such as professional and research background and family situation (eg, children). Our credibility comes from active listening and asking relevant questions which are meaningful to our participants [26]. 

### Study Design

The study uses a qualitative approach with topic-guided interviews and focus groups (Table 1). The qualitative content analysis approach according to Kuckartz will be used [27,28] and Charmaz constructivist grounded theory will be followed to develop a theory [29] of family life with children at risk of or diagnosed with FA.

**Table 1:** Study design.

Characteristics	Parent group	Health care professionals group
Participants	Parents of children (0-3 years) Diagnosed with a FA^a^ At risk of FA Without a known risk of FA	Health care professionals Pediatricians Allergists Pediatric dermatologists Gynecologists or obstetricians Midwives Nurses
Study design	Qualitative approach	Qualitative approach
Data collection	Topic-guided interviews Approximately 30 interviews	Topic-guided interviews and focus groups Approximately 15 interviews and 2-4 focus groups
Data analysis	Qualitative content analysis according to Kuckartz [27,28] Constructivist grounded theory according to Charmaz [29]	Qualitative content analysis according to Kuckartz [27,28]
Dissemination	Recommendations for the development of the NAMIBIO^b^ apps	Recommendations for the development of the NAMIBIO apps

^a^FA: food allergy.

^b^NAMIBIO: Nahrungsmittelallergie biomarker/food allergy biomarker.

### Theoretical Framework

Within the social constructivist perspective, we work with sensitizing concepts from the social sciences, for example, “doing family” [30-32], “health literacy” [33], “the social amplification and attenuation of risk framework” [34], “burden of illness” [35], “access to health care” [36,37], “peer support among patient communities” [38], and “guilt” [39].

These concepts are preliminary tools and repeatedly stimulate our thinking about the topic (eg, raising questions for the topic guides) and the generated data (eg, while analyzing processes and actions or interactions). We will dispense with particular sensitizing concepts, if they prove to be irrelevant [29].

### Participants

We focus on 2 different target groups—parents and HCPs: (1) The parent group includes caregivers (mothers or fathers) of children between the ages of 0 and 3 years diagnosed with FA, at risk of FA, or without a known risk of FA (Textbox 1). Participating parents receive 30€ (US $32.7) to compensate for their time and efforts. (2) The HCP group includes pediatricians, allergists, pediatric dermatologists, gynecologists or obstetricians, midwives, and nurses. Participation is remunerated with 80€ (US $87.2; Textbox 2).

**Inclusion criteria (parent group).**

- Parents of children (0-3 years) diagnosed with a food allergy
- Parents of children (0-3 years) at risk of food allergy
  Risk factors for food allergy in children
    Preexisting allergies or allergic diseases, for example, allergic asthma and hay fever
    Preexisting atopic eczema, especially one that starts early in life and is severe
    Genetic or environmental risk factors for food allergy, for example, food allergy, atopic eczema, allergic asthma, or hay fever in the family history (first generation)
- Parents of children (0-3 years) without a known risk of food allergy
- Living in Germany
- Written consent form

**Inclusion criteria (health care professionals group).**

- Health care professionals: Pediatricians, allergists, pediatric dermatologists, gynecologists or obstetricians, midwives, and nurses
- Working in an inpatient or outpatient setting in Germany
- Written consent form

### Recruitment Process

**Figure 1:**
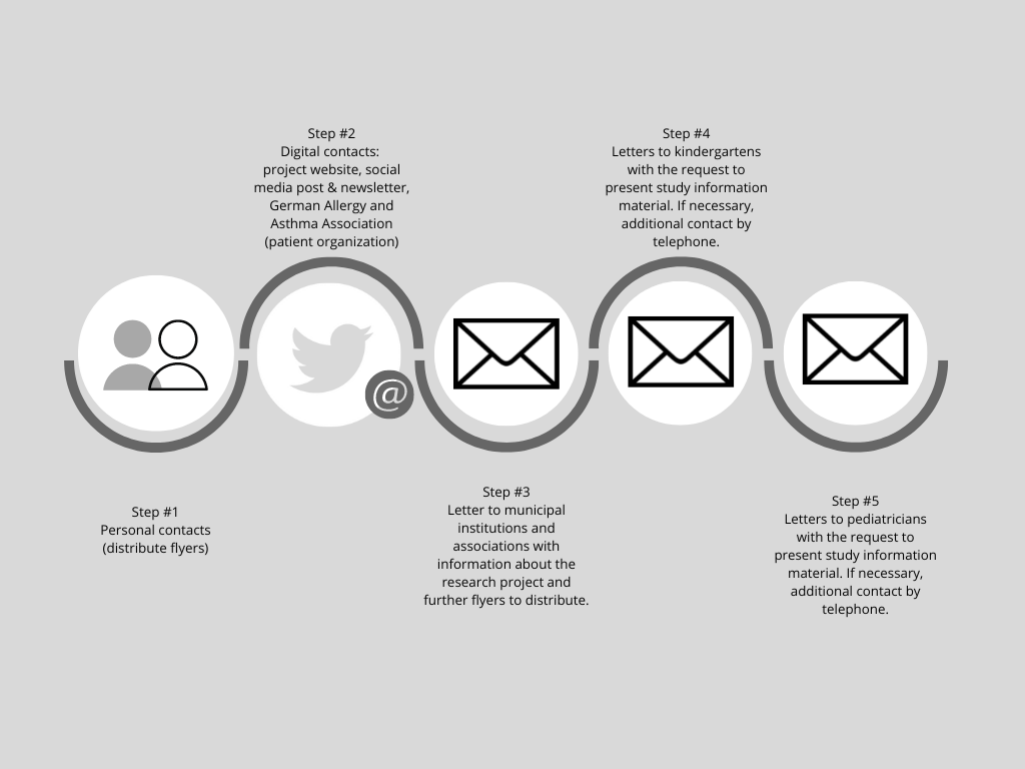
Recruitment strategy parents.

**Figure 2:**
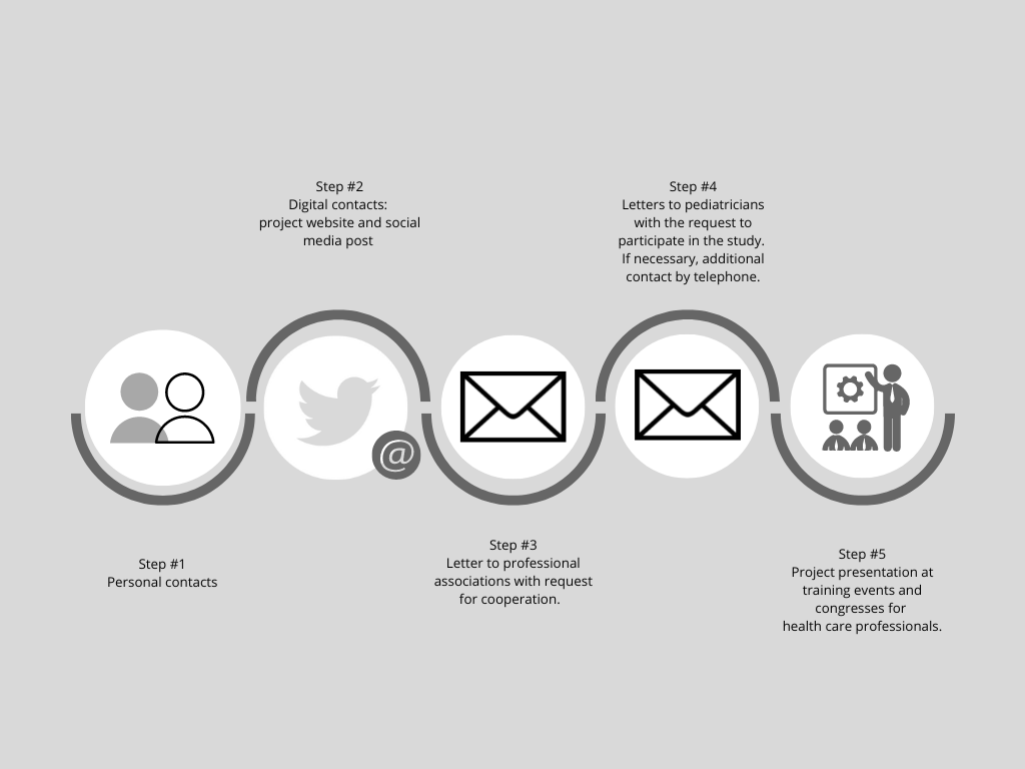
Recruitment strategy health care professionals.

Figures 1 and 2 display the strategies for the recruitment of participants for the parents and the HCPs group.

We first established field access through personal contacts (in our families and professional environment) at the 2 projects sites in Germany—Magdeburg and Northern Saxony-Anhalt and Regensburg and Eastern Bavaria—which differ in sociodemographic and structural characteristics [40]. It was ensured that there is no personal relationship between the interviewer and the interviewee.

Afterward, we recruited through a snowball sampling, followed by theoretical sampling.

The snowball sampling provides a point of departure to find relevant material for the study. This offers first insights into different (perhaps opposing) perspectives of the field, for example, parents of children without known risk of FA and parents of children diagnosed with FA or pediatricians and allergists. In addition, snowball sampling can prepare the theoretical sampling in a narrower sense [41]. The selection of further “cases” (theoretical sampling) is primarily concerned with the elaboration and contrasting of the phenomena found. Accordingly, the criteria for initial sampling may differ from those we use while sampling theoretically. The goal is to collect information from different perspectives in order to arrive at a vivid and comprehensive representation of the phenomenon of interest [42].

The size and composition of the 2 target groups (parents and HCPs) are not precisely defined. We anticipate about 30 interviews with parents, as well as about 15 interviews with HCPs and 2-4 focus groups. We aim for theoretical saturation of our data. “Saturation” means that collecting fresh data no longer leads to new theoretical insights and does not reveal new characteristics of the core theoretical categories [29].

### Setting

Interviews and focus groups are conducted in person, by telephone, or via privacy-compliant videoconferencing systems (Webex, Cisco), and in light of applicable COVID-19 regulations. Parent interviews are conducted individually (mother or father) or as a parent or family interview according to participants’ preferences.

### Data Collection

The topic guides were developed for the interviews with parents and focus groups [43] with HCPs (Multimedia Appendices 1 and 2). The questions were phrased openly to provide only as much guidance in the narrative flow as is needed [44] and yet support the interviewers in data collection. In the focus groups, case vignettes were used (Multimedia Appendix 2) to stimulate narratives at the beginning. The short presentations represent hypothetical case descriptions to foster discussion among the participants.

The German Allergy and Asthma Association (patient organization) provided feedback on themes and questions. We will adapt the guiding questions as needed during data collection, for example, to check hunches about categories, clarify relationships between emerging categories, or identify variations within a process (theoretical sampling) [29].

Audio data are recorded as mp3 files. To better contextualize the insights gained from the interviews and focus groups, parents’ sociodemographic data (year of birth, sex, nationality, marital status, country of birth, living situation, highest school or university degree, and employment or occupational status) as well as information on the HCPs’ career (qualification and professional experience) were gathered. In addition, field notes were written after the interviews and focus groups, which provide space for the researchers’ observations, perceptions, and further information.

### Data Analysis and Reporting

The interviews and focus groups are transcribed verbatim. MAXQDA Analytic Pro 2022 (VERBI software for qualitative and mixed methods research) is used to support us in managing and coding the data.

For answering our first research question, we will identify, conceptualize, and systematically describe relevant themes on parental information needs and seeking as well as health care usage and HCPs’ strategies on prediction and prevention of FAIC. A qualitative content analysis according to Kuckartz [27,28,45] will be applied. Kuckartz [27,28,45] describes different phases of data analysis that will be performed (Textbox 3). The procedure is rule governed and thus intersubjectively verifiable [27,45].

**Qualitative content analysis approach according to Kuckartz [27,45].**

Step 1: Preparing the data, initiating text work, including attentive reading and marking interesting parts of the text, noting special features and evaluation thoughts, and creating initial case summaries

Step 2: Forming main categories corresponding to the research questions

Step 3: Coding data with the main categories

Step 4: Compiling text passages of the main categories and forming subcategories inductively on the data material; assigning text passages to subcategories

Step 5: Category-based analyses and presenting results

Step 6: Reporting and documentation

The second research question (investigating the family’s experiences with children at risk of or diagnosed with FA) will be answered by using grounded theory methodology, which focuses on social processes and actions and interactions. Constructivist grounded theory [29] highlights shared meaning constructed by both participants and researchers. We use the 2 main types of coding of grounded theory: (1) initial coding, a strategy that helps us to conceptualize our ideas and (2) focused coding, which allows us to separate, sort, and synthesize our data. During initial coding, we study our transcripts line-by-line, (inter)action-to-(inter)action, or we adopt the narratives of our participants from time to time as in vivo codes [29]. Memoing on the interpretation and constant comparative analysis within and between interviews are used to confirm similarities and differences and push the analysis forward [29].

Weekly interpretative meetings including all researchers ensure an iterative process of data collection and analysis. Interpretations in groups are a discursive form (communicative validation) to establish intersubjectivity and comprehensibility [46,47]. At present, no respondent validation is planned.

Reportings will be based on COREQ checklist—consolidated criteria for reporting qualitative research [48]—that also guides the structure of this protocol.

### Ethics Approval and Data Protection

The ethics approval was obtained for the study from the Ethics Committee of the University Medicine Magdeburg (184/21). Participation in the interviews and focus groups is voluntary. Participants receive information about the aims and contents of the study as well as data protection at the time of initial contact and afterward by email or post. They provide written informed consent prior to participation. All study activities are conducted in strict compliance with the European Union’s General Data Protection Regulation and in accordance with the Declaration of Helsinki [49].

We are storing the data from the interviews and focus groups in a pseudonymized way. An independent trusted third party at the Medical Faculty of the Otto von Guericke University of Magdeburg manages data containing personally identifiable information (consent forms) and stores these data separately from the study data.

## Results

The project was funded from June 2021. Data collection started in February 2022. Depending on theoretical sampling, data analysis will continue until spring 2023.

## Discussion

The qualitative study will provide an in-depth insight into parents’ information-seeking behavior and needs regarding the prevention of FAIC, how parents use pediatric primary care, and what strategies HCPs’ use for prediction and prevention toward FAIC. The research team anticipates that the results will reveal challenges associated with the paradigm shift in allergy prevention for both parents and HCPs. We hope to be able to derive practical recommendations for the development of the NAMIBIO apps. Only by understanding the users’ perspective and the family’s experience with children who are at risk or diagnosed with FA, apps can be developed that correspond to the users’ everyday needs and accepted by them.

The results will be disseminated through scientific publications, social media (Instagram and Twitter), and at national and international conferences of health services research and allergy.

When we started the recruitment for our study, it was assumed that potential participants might have difficulties understanding the overall goal of the NAMIBIO app consortium. Since there is no direct objective-added value from participating in the study, we expected that it would be easier to recruit informed and engaged people to participate in the study. We assumed that mothers with a high level of education would be more likely to feel addressed in the recruitment process. In addition, we also anticipated difficulties in recruiting HCPs, especially because of both a lack of time and interest.

By now, we were able to recruit a diverse sample of parents and pediatricians in terms of age and personal or occupational experiences, capturing a variety of different perspectives. Our in-depth interviews and focus groups lasted up to 90 minutes. We followed all the steps of the recruitment strategies. Currently, it appears very difficult to recruit parents with a migration history and low level of education, especially fathers.

For this reason, the recruitment process is still ongoing. While we may not be able to overcome these challenges completely, we have launched a social media channel (Instagram: namibioapp) and ensured that we address organizations that have special access to the groups relevant to our study, in particular family and childcare institutions. Furthermore, we have adjusted the recruitment material. To gain better access to parents whose child has no known risk of FA, a broader approach focusing on general child health is used. In a preliminary contact, we inform about our study and ask about the risk factors for FAIC. In addition to German, the flyers are now also available in English, Turkish, Arabic, and Russian.

## Appendix

Multimedia Appendix 1 Topic guide for parents.

Multimedia Appendix 2 Case vignette and topic guide: health care professionals and focus group.

## Abbreviations

FA: food allergy
FAIC: food allergy in children
HCP: health care professional
NAMIBIO app: Nahrungsmittelallergie biomarker application (food allergy biomarker application)

## Data Availability

The data sets generated and analyzed during this study are available from the corresponding author upon reasonable request.
