# Supplementary material for: Parents’ and Health Care Professionals’ Perspectives on Prevention and Prediction of Food Allergies in Children: Protocol for a Qualitative Study
Source: JMIR Res Protoc. 2023 Mar 22;12:e41436. doi: 10.2196/41436 (PMC10131891; doi:10.2196/41436)
Supplement: Multimedia Appendix 1 [file resprot_v12i1e41436_app1.docx]

**Food Allergy Biomarker Application Consortium (NAMIBIO App)**

**Topic-guide for parents**

**Introduction**

At the beginning, I would like to thank you for your participation in the interview. You give us your important perspective on the early identification of risk factors and the early prevention of food allergies in children. We strongly believe that this is your contribution to raising awareness and possibly improving the prevention of food allergies (FA).

I am [name] and I am research assistant and member of the NAMIBO project team. Together with my colleagues at the project site in Regensburg/ Magdeburg, we are going to conduct interviews with parents as well as with pediatricians and allergists.

**Important notes:**

- Are there any further questions regarding the project and the consent form?

- There is no right or wrong - every individual perspective is important and valuable.

- The interview is recorded as an mp-3 file for data analysis. We are storing the data from the the interviews in a pseudonymised way.

- I would now like to start the recording.

**Introductory question - depending on whether we are interviewing parents of children diagnosed with FA, at risk or without a known risk of FA.**

*Diagnosis group:* Today I would like to talk to you about the topic food allergies in your child/children in your everyday family life. In doing so, I am interested in your personal perspective. I would therefore be pleased if you could tell me exactly how you are currently affected by the topic.

*Risk group:* Today I would like to talk to you about the topic to be at risk of food allergies in children in your everyday family life. In doing so, I am interested in your personal perspective. I would therefore be pleased if you could tell me exactly how you are currently affected by the topic.

*Without known risk group:* Today I would like to talk to you about the topic child health and prevention in your everyday family life. In doing so, I am interested in your personal perspective. I would therefore be pleased if you could tell me exactly how you are currently affected by the topic:

| Topic-guide | Prompts |
| --- | --- |
| ***Diagnosis & risk group: How do you experience everyday family life with a child at risk of food allergy or diagnosed with a food allergy?***   - When did you first become involved with the topic of nutrition and food allergies? - Are there other people with food allergies in your environment, e.g. friends or family members, or are you perhaps affected yourself? - How did you get the idea that your child might have a food allergy or be at risk for a food allergy? - When (with which symptoms) did you seek support/counselling? - What did all happened between the suspicion of a food allergy and the diagnosis?   ***Without a known risk group: When and how do you consider child health and allergy prevention in your everyday life?***   - How do you try to promote your child's health and prevent allergies? - Can you describe what you understand as ('allergy') prevention interventions? - When did you first become involved with the topic of nutrition and prevention of food allergies? - When (with which symptoms) did you seek support/counselling? | Previously you said that …  Did I understand correctly that ...  Can you explain that to me?  Can you tell me a bit more about this?  Can you give me an example?  Is there anything else?  Can you describe this in more detail?  Do you have an example of this so that I can imagine it more clearly?  What do you mean by that?  Who else was there?  When did X happen?  Where did X happen? |
| ***What information needs and requirements do you have with regard to the early prediction of risks and the prevention of food allergies?***   - Which information on prevention of allergies/food allergies are important and interesting for you? - How have you informed yourself about food allergies? - About what topic would you personally like to have more information/knowledge? |  |
| ***What strategies do you use to seek information and support?*** *^*^Parents of children with diagnosed food allergies are asked retrospectively*   - Where and how do you seek information? - What do you need to find information helpful? - Which information do you trust? - What helps you understand health information? - When do you decide to implement a recommendation in your family life? - How do you succeed in implementing knowledge from health information in everyday life? - Where and how do you get support on (prevention on) food allergies? |  |
| ***Do you use (electronic) decision support tools or information support applications for health issues?***   - Do you use and trust digital health applications, e.g. apps? - What do you think about using an app on food allergies in children? - How would you like to use this application? (App store/ web based) - When/ how might this be helpful for you? - We want to develop an app that can predict the risk for food allergie and offer targeted recommendations for prevention ... - What do you think about this? Would this (have been) helpful? Why (not)? - Which information would you disclose to an app? |  |

**Closing the conversation**

- Make it clear that the conversation is coming to an end.

E.g., from my side we have finished with the questions.

- Give the interviewee a concrete example of how his or her insights have made a contribution

E.g., we can use your personal experience on data protection directly in the app development.

- Ask the participant if there are any other questions that should be asked or topics that should be explored

E.g., we have now touched on many topics in the last xx minutes, is there anything else you want to get off your chest about the topic?

- Try to make new contacts

E.g., can you recommend other people I should talk to?

- Remind the respondent of possible follow-up conversations with the researcher or research team

E.g., we would like to get back to you once the (pilot) app is ready. Perhaps you might want to let us know your thoughts on it again then.

- Socio-demographic questions

Last point for today....

- Allowance for expenses
- Thank the interviewee once again
