# Supplementary material for: Parents’ and Health Care Professionals’ Perspectives on Prevention and Prediction of Food Allergies in Children: Protocol for a Qualitative Study
Source: JMIR Res Protoc. 2023 Mar 22;12:e41436. doi: 10.2196/41436 (PMC10131891; doi:10.2196/41436)
Supplement: Multimedia Appendix 2 [file resprot_v12i1e41436_app2.docx]

**Food Allergy Biomarker Application Consortium (NAMIBIO App)**

**Case vignette & topic guide: health care professionals - focus group**

**Case vignette**

Mrs Meyer has a three-year-old son. She reports that he did not have good skin since birth. At the U4 (preventative checkup for children between the 3rd and 4th month of life), neurodermatitis was diagnosed and she started to apply eczema cream. With the introduction of complementary feeding, she didn't notice anything at first until she started mixing butter or milk into the porridge. [Theo] was about six, seven, eight months old. "And that was when he didn't react so badly". She describes that she "somehow had a strange feeling. That he didn't really tolerate it". After a talk with the pediatricians, no further diagnostics were done. She should "try to leave it out and then see [...] if the skin improves."

When Theo was about one year old, he gets scrambled eggs for breakfast from his parents and "immediately starts to gag and cough and also vomited completely". Now it was "no longer a gut feeling, but that was really not cool". Mrs Meyer reports that the pediatricians then conducted an allergy test. Peanut, milk, egg, wheat and soy were positive. So she relatively quickly got them to "do the first food provocation to know if it was really severe or anaphylaxis". At that point, she also started to search Google for more information. Her husband also became a member of the DAAB to get more information and materials.

She talks about her initial worries - "no one tells you what you can give instead and how you can somehow make sure with such a small worm that it gets everything it needs. Then I asked our pediatricians if it was possible to get some kind of nutritional counselling, to get some kind of guide or something. And she said, "No, no, you do it already." Subsequently, nutritional counselling, arranged through the health insurance company, gave her a sense of security.

Looking back, Mrs Meyer reported, "Throughout the breastfeeding period-, well, I really ate loads of nuts. No wonder his skin looked like shit [...]. I think that would have been good to have some idea that it can affect you". Although Mrs Meyer has allergic asthma herself, she believes that her son's risk of food allergy would not have been that high.

Since she is beeing pregnant again, she has been concerned with the risk of food allergy in the unborn child. "The first time, I might have been clueless about it, but now it's getting exciting."

**Open questions:**

- What came into your mind while reading?
- How do you assess the situation?

**Topic-related questions:**

- What strategies do you use to predict and prevent food allergies in children?
- Which parents do you talk to about the risk of food allergy?
- What are the indications for diagnosis? How do you discuss risk with parents/children?
- How do you deal with uncertainties of risk prediction? How relevant is this to parents?
- What information regarding potential risk factors of food allergies are parents asked for?
- How/ in which cases do you cooperate with pediatricianss/ allergists/ nutritionists/ midwives?
- How do you discuss (risk of) food allergies in children with parents in your practice?
- How do you advise on emergencies?

**How do you inform yourself about food allergies and allergy prevention?**

**Which experiences do you have with (electronic) decision-making tools or applications for information support on health issues?**

- How often do you use and trust digital health applications in your professional practice?
- What do you think about (electronic) decision support or food allergy prediction and prevention apps?
- What would an app need to have in order to use it on food allergies or recommend it to families?
- When/ how could digital health apps on food allergies be helpful?
- How should an (electronic) decision support or food allergy information support apps be designed to meet your needs and those of parents?
- What do you need to recommend an app to parents?
